# Newly-Engineered Materials for Bio-Imaging Technology: A Focus on the Hybrid System of Ultrasound and Fluorescence

**DOI:** 10.3389/fbioe.2019.00088

**Published:** 2019-04-26

**Authors:** Yanbo Pei, Ming-Yuan Wei

**Affiliations:** ^1^Department of Physics, Harbin Institute of Technology, Harbin, China; ^2^Texas Commission on Environmental Quality, Fort Worth, TX, United States

**Keywords:** biomaterials, bioimaging, contrast agents, ultrasound-modulated fluorescence, ultrasound switchable fluorescence

## Abstract

As an emerging technique, ultrasound-modulated fluorescence (UMF), or ultrasound switchable fluorescence (USF) bioimaging has shown promising features to produce deep-tissue and high-resolution fluorescence imaging for biomedical research and health diagnosis. The success of UMF or USF heavily relies on the design of their contrast agents (CAs). We herein surveyed recent advances in the development of such unique CAs, including configuration, mechanism, stability, sensitivity, and selectivity. Meanwhile, UMF or USF instrumentation has emerged as developmental breakthrough technologies to existing bio-imaging techniques. The best performance of UMF or USF bio-imaging requires an interactive response between CAs and the instrument. In this review, the description of UMF or USF instrumentation are also included for clarification and better understanding. Finally, the UMF and USF's performance in bioimaging is evaluated based on signal-to-noise ratio, resolution, imaging depth and speed, using photoacoustic imaging (PAI) as a standard, a well-developed technique of hybrid bio-imaging. Unlike PAI, UMF or USF is still in its early stage. Although results demonstrated a proof-of-concept landmark being reached, significant efforts are needed to improve the performance of UMF or USF.

## Introduction

Fluorescence imaging is a procedure widely used in biomedical laboratories for preclinical experiments and clinical stages, due to the rapid development of optical technology and the large number of fluorescent probes capable of emitting various wavelengths for targeting a variety of specific biological structures (Salzer, 2012). One of the advantages of fluorescence imaging is its ability to provide a variety of information about the structural, physiological and molecular structure of a cell or tissue. Fluorescence imaging is also widely used because the near-infrared light used for imaging is a non-ionized and non-radioactive radiation. Fluorescence imaging methods can be divided into two categories, based on the target image size and the target image depth. The first method is to image the target through a microscope. Fluorescence microscopy requires that the imaging target be a cell or a thin tissue with a thickness (depth) of ~1 mm or less. The resolution has the capacity of capturing the diffraction limit of the corresponding wavelength of light. The second type of method is macroscopic imaging based on optical tomography. The imaging depth of this method extends to several centimeters; however, the resolution depends on the imaging depth. Therefore, the resolution varies; for small animal imaging, the resolution can be in millimeters, but imaging resolution of larger tissues, such as human breasts, is limited to centimeters.

Although the fluorescence imaging method has advantages, the aforementioned imaging methods do not meet the current requirements of deep-tissue and high-resolution for biomedical imaging, especially *in-vivo* imaging. Although the microscope has a high resolution, it can only perform surface imaging. Photons emitted or scattered by an imaging target with a depth of more than 1 mm will be scattered multiple times so that the image becomes blurred (Miller et al., 2017). The optical tomography method uses scattered photons to produce images (Corlu et al., 2007; Solomon et al., 2011). The microscope can produce images up to centimeters in depth; however, the resolution of these images is greatly reduced. Procuring images of deep tissue in high-resolution fluorescence imaging is a very important and extremely challenging problem in the field of biomedical imaging, which still requires work to be addressed.

When imaging a target with at a depth of more than 1 mm within a biological tissue, the scattering of photons used for imaging throughout the tissue results in reduced resolution. Ultrasonic waves with frequencies in megahertz are scattered two to three magnitudes smaller than of photons (Salzer, 2012). The current ultrasound imaging system can achieve imaging depths of up to 9 cm in B mode, but resolution levels of 1 mm are limited to a depth of 5 cm. It is envisaged to combine the large depth and high resolution from ultrasound imaging, including a variety of other information and strong contrast from fluorescence imaging, in order to achieve large-depth and high-resolution fluorescence imaging in biological tissue—a hybrid imaging mode combining ultrasound and fluorescence.

In 1995, observations of the optical signal modulated by ultrasound were reported (Leutz and Martet, 1995; Wang et al., 1995). Biological tissue was illuminated using coherent light, and focused ultrasound modulated the propagation of light at the focal point, which caused vibration of the speckle on the surface of the tissue. The optically modulated signal of the surface speckle reflects the optical properties of biological tissue through ultrasound focal volume. In 2006, ultrasound-modulated fluorescence (UMF) observation was first reported, which laid the foundation for the development of UMF tomography (Kobayashi et al., 2006). In 2012, two research groups independently reported the use of an imaging mechanism based on ultrasound switchable fluorescence (USF) (Lin et al., 2012a,b; Yuan et al., 2012). These progressive developments began to harness an exciting future for deep-tissue and high-resolution fluorescence imaging.

The well-developed photoacoustic imaging (PAI), also known as optoacoustic imaging or thermal acoustic imaging, is now dominating the field of optics/ultrasound hybrid bioimaging (Upputuri and Pramanik, 2016; Wang and Yao, 2016). As emerging technologies, UMF and USF are still in their initial 10-year stage. Yet both have shown great potential to improve the quality of deep-tissue and high-resolution bioimaging, as a result of combining the advantages found in ultrasound (resolution and depth) and fluorescence (contrast or sensitivity) (Kobayashi et al., 2006; Lin et al., 2012a; Yuan et al., 2012). Fluorescence is used as the signal generation of UMF and USF, rather than heat (as recorded in PAI), which allows UMF and USF to utilize the versatility of fluorescence imaging technology, such as lifetime imaging, quenching, and Förster (or Fluorescence) Resonance Energy Transfer (FRET). More importantly, the success of UMF and USF highly depends on the design of the contrast agents (CAs). As discussed earlier, due to the background noise of scattering light in tissue, the optical window for bioimaging is in the range of near-infrared (NIR) wavelengths. Fluorophores that have NIR emission have been widely used as CAs for bioimaging (Guo et al., 2016). For instance, indocyanine green (ICG), whose emission wavelength is at approximately 830 nm, was approved by the U.S. Food and Drug Administration (FDA) for bioimaging application^1^. CAs using fluorescent nanomaterials (Hahn et al., 2011; Wolfbeis, 2015; Reineck et al., 2017) show more promising features in comparison to organic fluorophores, such as better biocompatibility, stability, the ease of functionalization, and the capability of simultaneous multiple targets analysis thanks to the narrowed emission spectrum (e.g., quantum dots). Several nanomaterials showed NIR fluorescent emission: (Reineck et al., 2017) quantum dots, polymer dots, carbon nanotubes, or upconverting nanoparticles. The other way to eliminate the fluorescence background from tissue is fluorescence life-time based bioimaging. The lifetimes of autofluorescence in tissue are in the range of 0.1–7 nanoseconds (ns), (Berezin and Achilefu, 2010) thus the life-time of CAs needs to be close to or longer than 10 ns. Quantum dots (lifetime: 10–30 ns) or upconverting nanoparticles (lifetime >100 μs) is a potential candidate for CA development. We summarized the recent advance in the development of UMF and USF CAs. An overview of the development of CAs can be found in Table 1. Note that the development of CAs for PAI has been widely researched and tested over decades, and review articles are readily available (Jiang and Pu, 2017; Zeng et al., 2018). From Table 1, one could find that most of the CAs for USF or UFM are exogenous agents due to the special conditions of NIR fluorescence emission. To date, UMF or USF CAs were limited in using fluorophore as the signal generator. Nanomaterials or micro-particles were reported to serve as a carrier to attach the fluorophore molecules on the surface or load them inside. The mechanism of UMF is still under debate, and more details will be seen in the following section. As for USF, heat provided by ultrasound resulted in the reformation of the nanomaterial (the carrier); the micro-environment inside the nanomaterial changed, for instance, from hydrophilic to hydrophobic, leveraging the quantum yield of the fluorophore; the fluorescence intensity was therefore enhanced (turned “ON”). The carrier of nanomaterial ensures the switch of fluorescence from OFF to ON, but also provides the ease of functionalization for anchoring targeting moieties, such as antibody or receptor. As we describe the design and features of unique CAs for UMF and USF, we also address the following as an effort to ensure clarity and understanding: imaging mechanism, imaging performance, and comparison with PAI.

**Table 1:** Overview of contrast agents for UMF, USF, and PAI.

		**UMF**	**USF**	**PAI**
Endogenous		NR	NR	Jiang and Pu, 2017; Zeng et al., 2018
Exogenous	biomolecules	NR	NR	Upputuri and Pramanik, 2016; Wang and Yao, 2016; Jiang and Pu, 2017; Zeng et al., 2018
	organic dyes	(Lin et al., 2012b; Liu et al., 2014)	NR	
	inorganic	(McNeil, 2009; Miller et al., 2017)	NR	
	dyes@polymer nanocomposites	NR	Yuan et al., 2013; Cheng et al., 2014, 2016, 2017; Pei et al., 2014; Yu et al., 2016	
	dyes-labeled polymer	NR	Kobayashi et al., 2006; Cheng et al., 2014	NR
	dyes/microbubble	(Loving et al., 2010; Lin et al., 2015; Liu et al., 2015)	NR	NR
Functioning mechanism		Under debate	Ultrasound → Heat → phase change → Microenvironment → Quantum yield (dye) increases → Fluorescence “ON”	Absorb photons → heat
stability		NR	>6 months (Yu et al., 2016)	NR
			>10 months (Cheng et al., 2016)	
Sensitivity (switchable)		No	Yes	Yes
Selectivity (targeting)		Yes^*^	Yes	Yes
Versatility of detection		Yes	Yes	No
Commercial availability		Yes	No	Yes

*NR, not yet reported; FRET, Förster (or Fluorescence) Resonance Energy Transfer*.

* *limited due to the size of microbubble*.

## Ultrasound Modulated Fluorescence (UMF)

### UMF CAs

**Figure 1:**
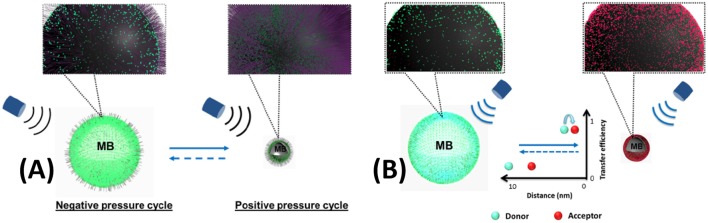
UMF contrast agent using microbubble as a carrier and a mediator. **(A)** single fluorophore-labeled microbubble (Liu et al., 2014) **(B)** donor- and acceptor-labeled microbubble (Liu et al., 2015). Reproduced with permission. Copyright 2014 & 2015^©^SPIE.

In first report of UMF, an organic fluorophore was used as the CA (Kobayashi et al., 2006). Subsequently, a report detailed the investigation of creating UMF signal on fluorophores attached to the surface of a microbubble. Fluorescence quenching is strongly related to the intermolecular distance of dye molecules (Geddes, 2001). A microbubble will be compressed under the positive pressure of ultrasound, while it is expanded under negative pressure. The intermolecular distance of the surface-attached dye molecules will decrease as the microbubble is compressed. This leads to fluorescence quenching and to a decrease of fluorescence intensity. Therefore, fluorescence quenching can be observed on a dye-labeled microbubble and modulated by ultrasound (Figure 1A). A microbubble was employed as a carrier for loading dyes on its surface and as a mediator to control the intermolecular distance of dye molecules. Likewise, FRET efficiency highly depends on the intermolecular distance of the donor and acceptor (Jares-Erijman and Jovin, 2003). While a pair of FRET donor/acceptor were attached on the surface of a microbubble, FRET was modulated by ultrasound, as demonstrated in Figure 1B.

### Typical Ultrasound-Modulation Fluorescence System and Acousto-Fluorescence Tagging Mechanisms

**Figure 2:**
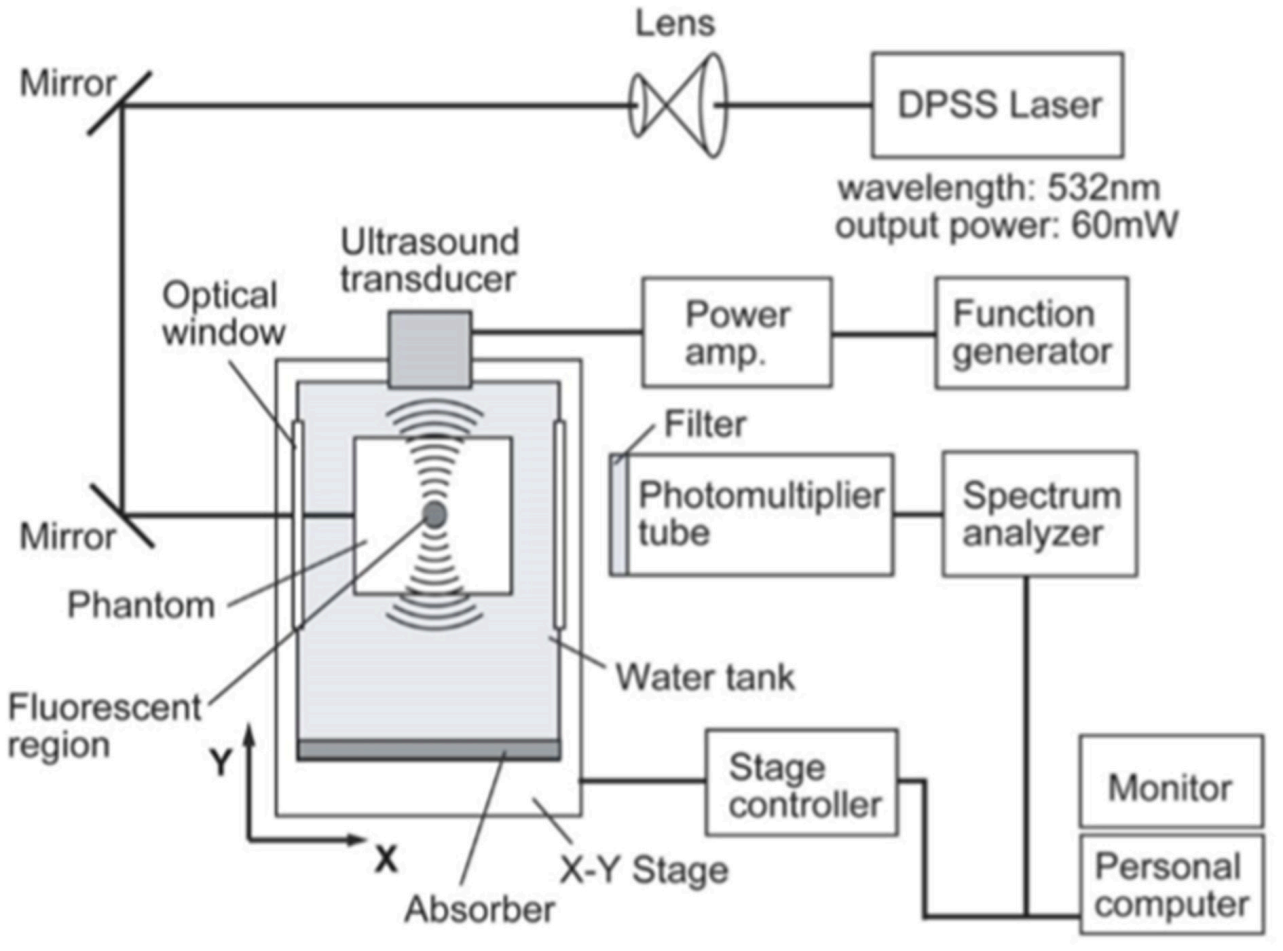
Typical ultrasound-modulated fluorescence imaging system (Kobayashi et al., 2006). Reproduced with permission. Copyright 2006^©^API Publishing.

UMF is based on the principle of sound vibration modulating the fluorescent emission of the fluorophore in the sound field, and therefore when the fluorophore is in the scattering medium, it can be imaged by scanning the focused sound field. Shown in Figure 2 is a typical UMF imaging system. In the first report on UMF in 2006, it was inferred that the fluorescence modulation is due to changes in the refractive index and scattering coefficient of the medium in the sound field (Kobayashi et al., 2006). If the tissue containing fluorescent dye is in the sound field, a change in the density of the material caused by the sound field will result in a change in the density of the photons. Therefore, a change in the refractive index and the scattering coefficient caused by the sound field will cause a deflection of the propagation path of the light, which then causes modulation of the fluorescence intensity. Here, the modulation depth of the fluorescence signal is a quadratic dependent on the sound pressure. From this, other researchers often used the dependence of fluorescence modulation depth on sound pressure as an important evidence for discerning fluorescence modulation mechanisms.

In 2008, researchers reported that the UMF signal was mainly due to changes in the concentration of the fluorophore caused by sound field vibration (Yuan et al., 2008). In regards to the concentration and the loading method of the fluorophore, imaging mechanisms using fluorophore can be divided into two categories. The first type of mechanism occurs when the concentration of the fluorophore is so low that the quenching effect of the fluorophore is negligible. When this occurs, the UMF signal is mainly attributed to the modulation of the fluorescence emission intensity reflecting the fluorophore concentration modulation, which is caused by ultrasonic vibration. This results in a much weaker modulation of fluorescence due to the change in the refractive index and the scattering coefficient of the material with the ultrasonic vibration. The second type of mechanism occurs when the concentration of the fluorophore is high. At high concentrations, ultrasonic vibrations cause volumetric compression, which results in significant fluorescence quenching or FRET. Under these two mechanisms, the effect of using microbubbles as contrast agents is more obvious (Yuan et al., 2009; Yuan and Liu, 2010). Because the microbubbles can produce greater volumetric compression and expansion changes as they vibrate in the sound field, the concentration of the fluorophore alters more sharply. In the above model, the first mechanism expects that the fluorescence modulation depth will be linear with the sound pressure of the sound field; and in the second mechanism, the fluorescence modulation depth will be non-linearly related to the modulation of the sound pressure. Therefore, some believed that the mechanism of UMF in the pioneering work by Kobayashi et al. has contributed the understanding of both fluorophore concentration changes and chromophore fluorescence quenching due to the expansion and compression of fluorescent spheres caused by the sound field (Kobayashi et al., 2006). These findings related to the dependence of fluorescence modulation depth on sound pressure has been supported by the subsequent experimental results.

So far, the mechanism of ultrasound-modulated fluorescence imaging has not been fully researched and understood. In 2010, researchers discussed the mechanism of ultrasound-modulated fluorescence based on experimental findings (Yuan and Liu, 2010). It was further discussed that in the case of low concentration of fluorophores, the mechanism of ultrasonic modulation of fluorescence originates from the modulation of the luminescent properties of the fluorophore by the sound field, such as concentration, quantum yield, and fluorescence lifetime. Unfortunately, which luminescent property is modulated has not yet been distinguished.

### Performance of Ultrasound-Modulation Fluorescence Imaging

In the first report on UMF in 2006, Kobayashi et al. used continuous ultrasound at a frequency of 1 MHz, with a focal point diameter of 3 mm (Kobayashi et al., 2006). The sound pressure at the focus of the sound field is 4.1 × 10^4^ Pa. The imaging target was a 5 mm long and 3 mm diameter column containing fluorescent microspheres embedded in a 40 × 40 × 75 mm^3^ agar biological tissue phantom. The authors used the focus of the ultrasound to scan two-dimensionally along a section of the cylinder perpendicular to the symmetric axis. The resulting image has a full width at half maximum (FWHM) of 3 mm. A very good signal strength was achieved in this study (the maximum signal strength on the fluorescent target is about 10 times that of the background signal), although this was obtained when the size of the imaging target was large.

In 2008, researchers who had based UMF mainly on the modulation of fluorophore concentration by the sound field, began to study the theory of the imaging mode of ultrasonic modulation fluorescence (Yuan et al., 2008). Theoretical results showed that when the size of the ultrasound focus was reduced to about 1 mm, the ratio of modulated fluorescence to unmodulated fluorescence in the modulation region was between 10^−4^ and 10^−6^. This ratio is lower if one considers that the fluorescence outside the modulation region actually contributes the unmodulated fluorescence a lot. Therefore, the fluorescence intensity modulated by the ultrasound is very weak relative to the intensity of the unmodulated fluorescence.

In 2009, researchers proposed using microbubbles as contrasting agents to enhance the fluorescence signal of ultrasound modulation (Yuan et al., 2009). They used a silicone tube with a diameter of 0.79 mm as the imaging target, and the silicone tube was filled with a fluorophore solution in which microbubbles 2.5 μm in diameter were dispersed. The depth of the silicone tube in the biological tissue phantom was 2 cm, and the lateral full width at half maximum of the ultrasound focus was 2.3 mm. The ultrasonic modulated fluorescent signal profile of the silicone tube obtained had FWHM of about 2.3 mm. In particular, they emphasized the comparison of a fluorophore solution, a mixture of fluorophore and microbubble for producing the UMF image profile of a mixture in a silicone tube. Through this comparison, it was found that the contrast of the latter was nine times than that of the former two.

**Figure 3:**
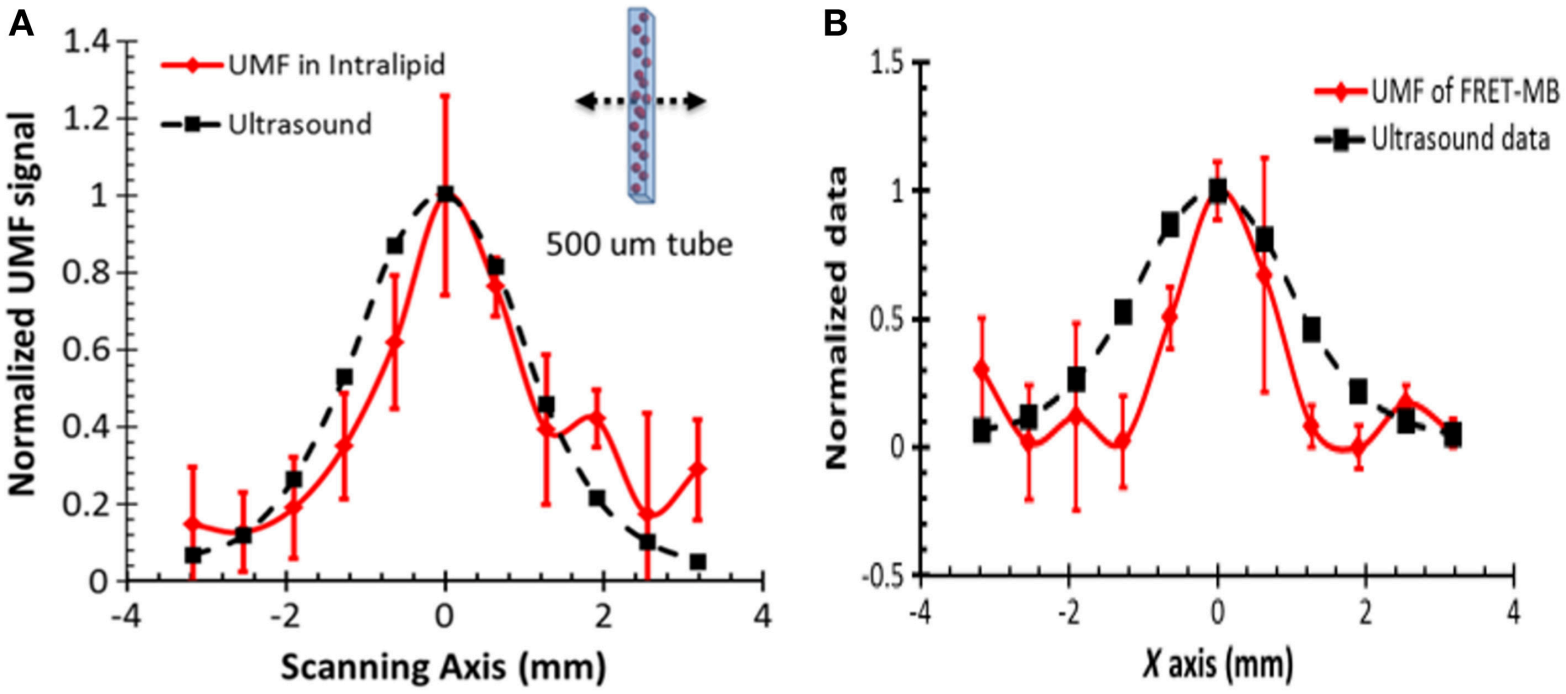
Normalized UFM signal from fluorescent microbubbles filled in a microtube through a 2-mm-thick intralipid slab phantom as a function of the lateral location of the 1-MHz ultrasound transducer. The dotted square line shows ultrasonic echo data that were recorded based on the conventional pulse-echo method. The solid diamond line shows the UMF signal. The arrow in the inset indicates that the transducer was scanned across the tube repeatedly for three times. **(A)** A single type of dye was attached to the surface of the microbubble and a significant self-quenching effect occurred when no ultrasound was applied (Liu et al., 2014) **(B)** Microbubbles were simultaneously labeled with donor and acceptor fluorophores on the surface to minimize self-quenching and maximize FRET (Liu et al., 2015). Reproduced with permission. Copyright 2014 & 2015^©^SPIE.

In 2014, researchers used a single fluorophore-labeled microbubble as a CA for UMF (Liu et al., 2014). When microbubbles are in a natural state, the density of the fluorophores marked on the surface is high and are in a severe fluorescence quenching state. When a microbubble undergoes a volumetric compression and expansion change under the action of the sound field, a change in the surface fluorescence chromophore concentration causes a change in the surface fluorescence quenching state, thereby resulting in sound field modulation of the emitted fluorescence intensity. In their work, they observed that a single microbubble was driven by the sound field to achieve a 42% fluorescence modulation depth. In a 2 mm deep biological tissue phantom, the ultrasonically modulated fluorescence image of a 0.5 mm microfluidic channel has a FWHM of 2 mm (Figure 3A), which is approximately the diameter of the ultrasound focus. In 2015, the same group used the donor and acceptor chromophores to label microbubbles, resulting in FRET occurring between the donor and the acceptor (Liu et al., 2015). The vibration of the microbubbles in the sound field will modulate the strength of the resonance energy transfer, thereby realizing the modulation of the fluorescence intensity of the donor and acceptor molecules. A single microbubble achieved a modulation depth of up to 33%. The silicone tube had a depth of 2 mm and a diameter of 0.5 mm, and the contour of the UMF image in the biological tissue had a FWHM value of approximately 2 mm (Figure 3B).

### New Ideas to Potentially Improve the Imaging Performance

Ultrasound-modulated chemiluminescence (UMCL) eliminated the scattering light background from the excitation light, which may be beneficial to improve the signal-to-noise ratio. To date, the bioimaging performance of UMCL and UFM appears comparable. More efforts are needed to improve the bioimaging performance of UMCL, yet we believe the idea may shed light on the development of UMF bioimaging. In 2013, researchers attempted to use luminescent materials as imaging targets to study ultrasound-modulated luminescence imaging in biological tissue phantoms (Huynh et al., 2013). The study used a 1 megahertz ultrasound to image the luminescent target with a diameter of 1 mm and a length of 5 mm at a depth of 7 mm in the biological tissue. Since the imaging target is not on the focus, the image has a FWHM of 6 mm. This imaging mode is expected to have a high signal to noise ratio, since no external excitation light is required. Researchers employed numerical methods to study UMF imaging of targets containing luciferase in biological phantom (Zhang et al., 2015). They achieved an imaging resolution of 3 mm at a 7 mm-depth with a signal-to-noise ratio of 80.

## Ultrasound Switchable Fluorescence

### USF CAs

As shown in Table 1, the functioning mechanism of USF CA is a triple-transition process: (1) high intensity focused ultrasound (HIFU) provides heat and raises local temperature; (2) the internal micro-environment changes through a phase transition (i.e., heat), lowering polarity and becoming more hydrophobic; (3) the quantum yield of a fluorophore increases upon the decreased polarity, resulting in an enhancement in fluorescence intensity or lifetime. Thus, the detection turns “ON”. It is in the “OFF” status that the background fluorescence is negligible without HIFU irritation.

**Figure 4:**
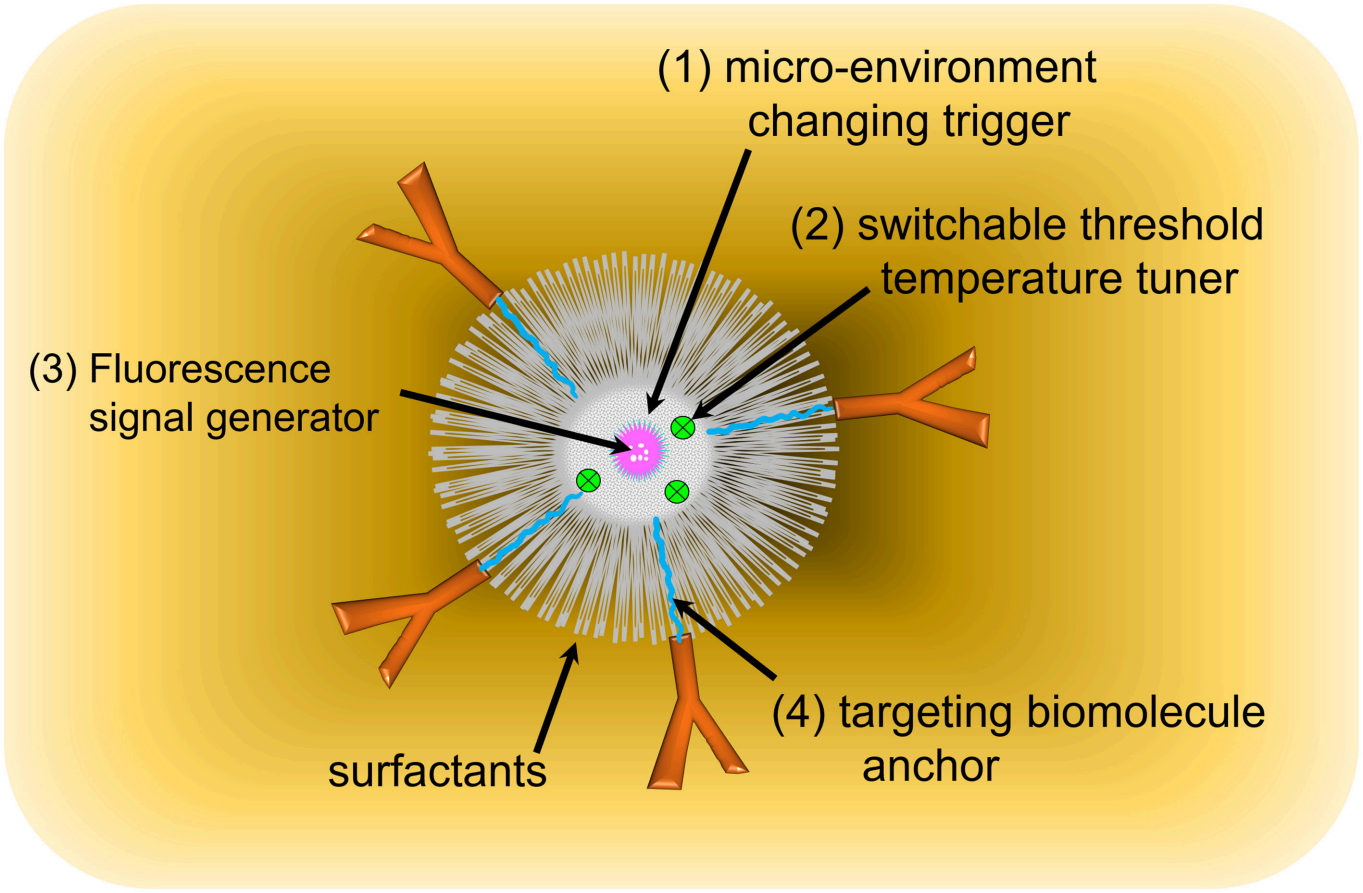
USF contrast agent's components.

Four key components are essential to designing a USF CA, as seen in Figure 4: (1) A micro-environment changing trigger. A thermo-responsive polymer, micelle, or nanocomposite was selected to meet this requirement. Poly(N-isopropylacrylamide) (PNIPAM) or Pluronic polymer has shown excellent phase transition for triggering temperature (Pei et al., 2014; Cheng et al., 2016), providing a switch-like response. (2) A switchable threshold temperature tuner. Hydrophilic or hydrophobic moieties were conjugated to PNIPAM or Pluronic polymer, and the resulting polymer's switchable threshold temperatures are either increased or decreased. (3) A fluorescence signal generator. A micro-environment-sensitive fluorophore was selected to generate the “ON” fluorescence emission signal due to the local microenvironment changes (Baruah et al., 2006; Loving et al., 2010). (4) A targeting biomolecule anchor, which are often functional chemical groups, such as amine, carboxyl, and hydroxyl groups. They allow the targeting biomolecules, such as antibodies, receptors, or DNA/RNA hairpins, to be attached to the CAs through chemical conjugation, electrostatic force, or bio-affinity reaction. Additionally, a stabilizer, such as SDS or other surfactants, is included to prevent aggregation of nanocomposites or micelles. Detailed information about targeting biomolecules and surfactants was provided in the following discussion.

The development of USF CAs began with using linear PNIPAM polymers labeled with a fluorophore, namely N-{2-[(7 -N,N-dimethylaminosuflonyl)-2,1,3-benzoxadiazol-4-yl](methyl)amino}ethyl-N-methylacrylamide (DBD-AA) (Yuan et al., 2012). A series of similar linear CAs were investigated, and details about the design and functioning mechanism of such CAs can be found in our previous review article (Cheng et al., 2014). Linear-polymer-based CAs were standardized using five parameters: (1) peak excitation and emission wavelengths (λ_ex_ and λ_em_); (2) the fluorescence intensity ratio between on- and off-states (I_On_/I_Off_); (3) the fluorescence lifetime ratio between on- and off-states (τ_On_/τ_Off_); (4) the temperature threshold to switch on fluorophores (T_th_); and (5) the temperature transition bandwidth (T_BW_). Results substantiated a proof-of-concept for USF CAs. However, linear USF CAs often lack biocompatibility and were not a suitable candidate for practical bio-imaging. Nanocomposites or micelles made of PNIPA or Pluronic polymer were synthesized, in which fluorophore molecules were attached on surface or encapsulated, as seen in Table 2.

**Table 2:** Overview of components of USF contrast agents.

		**linear polymer**	**polymer nanocomposite**		**micelle**
Single fluorophore	DBD; St633; Sq660; St700; ADP-CA; ICG	PNIPA	PNIPA		Pluronic F-98; F-127
Donor and acceptor (fluorophores)	DBD/ St660	PNIPA	PNIPA	Surface	NR
	DBD-ED /Sq660		Core-to-surface		
Switchable threshold temperature tuner (co-polymers)		TBAm; AAm; AH; AAc	TBAm; AAm; AH; AAc		PEG-20K/ -30K/-40K
Targeting biomolecule anchor (functional groups)		AAc(carboxyl); AH(amine)	AAc(carboxyl); AH(amine)		NR
Others (surfactants)		n/a	SDS; F-127 (-carboxyl); F-98		PEG

**Figure 5:**
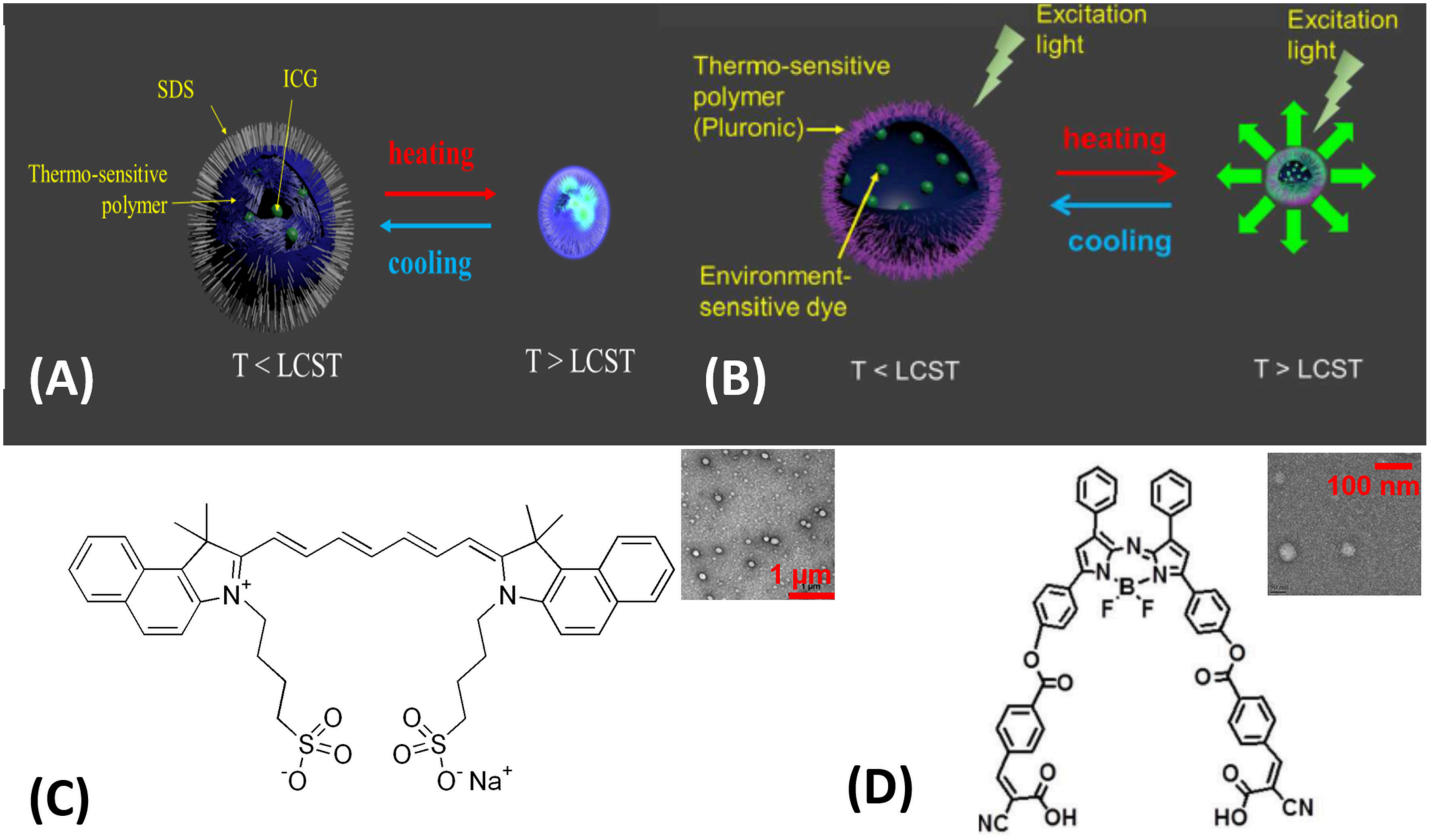
Two classes of USF contrast agents: **(A)** ICG-encapsulated PNIPAM NPs (Pei et al., 2014). Thermo-sensitive polymer: PNIPAM; environment-sensitive dye: ICG. **(B)** ADP-CA-encapsulated Pluronic micelle (Cheng et al., 2016). Thermo-sensitive polymer: Pluronic polymer; environment-sensitive dye: ADP-CA. Chemical structures of ICG **(C)** and ADP-CA **(D)**. LCST: lower critical solution temperature. Insert **(C)** Transmission electron microscopy (TEM) image of **(A)**, scale bar: 1 μm; Insert **(D)** TEM image of **(B)**, scale bar: 100 nm. Reproduced with permission. Copyrights 2014©Springer Nature and 2016©PLOS.

*DBD-ED, [7-(2-Aminoethylamino)-N,Ndimethyl-4-benzofurazansulfonamide; ICG, indocyanine green; ADP-CA, chemical structure see Figure 5D; PNIPAM, poly(N-isopropylacrylamide); TBAm, N-tert-butylacrylamide; AAm, acrylamide; AAc, acrylic acid; AH, allylamine; SDS, sodium dodecyl sulfate; PEG, Polyethylene glycol; NR, not reported yet*.

The nanocomposite-based CAs showed a comparable performance to the linear CAs (Cheng et al., 2014). PNIPAM nanocomposite were intensively studied as the model drug delivery carrier, due to its sharply thermos-responsive property. Instead of small-molecule drugs, micro-environment-sensitive fluorophores were encapsulated into a PNIPAM nanocomposite (Figure 5A). ICG dye has a near infrared emission (~810 nm), great water-solubility, and most importantly, it demonstrates an enhanced fluorescence intensity upon micro-environment changes (Pei et al., 2014) (e.g., decreasing polarity or increasing viscosity). ICG-encapsulated PNIPAM nanocomposites were synthesized *via* free-radical polymerization in a water phase where ammonium persulfate (APS) served as an initiator and tetramethylethlyenediamine (TMEDA) served as an accelerator (Pei et al., 2014) Sodium dodecyl sulfate (SDS), a surfactant, was also added into the polymerization reaction mixture to form nanocomposites. ICG solution was pre-mixed with the polymerization solution, and ICG molecules were encapsulated inside the nanocomposite due likely to ICG molecule's ampliphic property (see chemical structure in Figure 5C). The resulting ICG-encapsulated PNIPAM nanocomposite has a diameter at approximately 100 nm, see the representative TEM image in the insert of Figure 5C.

ICG-encapsulated PNIPAM nanocomposites showed a fluorescence intensity ratio between on- and off-states (I_On_/I_Off_) of approximately three (Pei et al., 2014). The ratio was found to undergo no significant change during twelve repeating ON/OFF measurements. It appeared that ICG molecules were not rapidly released from the nanocomposites because the ICG's molecular weight (774.96 Dalton) was larger than the recommended fast-release limit of small molecule drugs (500 Dalton) and close to the maximum limit (900 Dalton). Therefore, the nanocomposite was chosen as the first USF CA (No. 1) for further bioimaging study.

By conjugating with TBAm (hydrophobic) or AAm (hydrophilic), the switchable threshold temperature of the USF CA No.1 (~36°C) could be tuned either lower or higher (Pei et al., 2014). For instance, with a combination of 86 NIPAM/14 AAm in molar ratio, the switchable threshold temperature was adjusted to ~41°C, which is in the range to detect a cancer/tumor cell that often has higher temperature than normal cells. Furthermore, it is of the ease to conjugate functional monomers, such as AH (amine) or AAc (carboxyl), to the class of USF CA No. 1 for future targeting bioimaging application.

The shelf-life of the class of USF CA No.1 was unfortunately <30 days, as a result of the oxidation between ICG dye and APS or TMEDA (Yu et al., 2016) during the polymerization reaction. A slight change has been made to avoid the oxidation by using 4-4'-azobis(4-cyanopentanoic acid) (ACA) as the initiator for the polymerization reaction. The modified USF CA No.1 demonstrated an excellent shelf-life of 6 months (Yu et al., 2016). Another consideration is given to the toxicity of the short-length molecule surfactant SDS. SDS was replaced by long-chain surfactant Pluronic F-127 or F-98 (Yu et al., 2016), which yielded a better biocompatible USF CA No. 1. It is noted that the toxicity/biocompatibility of nanomaterials is a complex issue (Hahn et al., 2011). The main consideration is the size, surface charge, and solubility of the nanoparticle in response to uptake and clearance, cytotoxicity, and RES recognition (McNeil, 2009). A comprehensive evaluation of USF CAs may be provided in future studies. The hydroxyl group at the terminal of Pluronic surfactants was modified to a carboxyl group through a reaction with succinic anhydride. The modification allows further conjugation with bio-recognition moieties to the USF CA No.1.

The continued search for micro-environment-sensitive fluorophore resulted in a new finding. ADP-CA's quantum yield showed extraordinary enhancement when the polarity decreased, because a hydrophobic micro-environment mitigates the intramolecular quenching (Cheng et al., 2017). The intramolecular quenching is likely attributed to the internal photon-induced electron transfer from the benzene moieties to the aza-BODIPY core (Figure 5D). Due to its poor water solubility, it was challenging to integrate ADP-CA into our previous synthesis protocol for ICG. As a result, a protocol to apply oil/water phase transfer was adopted to form an ADP-CA-encapsulated micelle (Cheng et al., 2017), as shown in Figure 5B. Pluronic F-98 was chosen as the thermos-responsive polymer to synthesize the micelle. The resulting ADP-CA-encapsulated Pluronic micelle was namely USF CA No. 2. The average size of these micelles is in the range of 35–70 nm, see the representative TEM image in the insert of Figure 5D.

The USF CA No. 2 showed an impressive I_On_/I_Off_ in a range of 200~400, over 60 times higher than that of USF CA No. 1. By adding PEG-modified F-98 into the micelle, the switchable threshold temperature was adjusted from 30 to 45°C (Cheng et al., 2017). As mentioned above, functionalization of the USF CA No.2 could be obtained through converting hydroxyl to carboxyl. Additionally, the shelf-life of the USF CA No.2 was found to be longer than 10 months.

Performance basing on FRET was evaluated on USF CA. No. 1, which can be seen in Table 2. Two configurations were investigated: (1) both surface donor and acceptors, and (2) one of the two inside nanocomposite and the other on surface. While the latter demonstrated a better performance (Cheng et al., 2014), the lifetimes of the acceptor were found to be in the range of a few nanoseconds (ns), which is similar as that of autofluorescence in tissue (0.1–7ns) (Berezin and Achilefu, 2010). To eliminate the autofluorescence background, the lifetime of USF CA needs to be close to or longer than 10 ns. Organic dyes with fluorescence lifetime above 10 ns often fall into blue light or ultraviolet wavelength (Berezin and Achilefu, 2010). Quantum dots (QDs) have long fluorescence lifetimes (average 10–30 ns, up to 500 ns) and NIR excitation/emission wavelengths, which could make them promising FRET donors for lifetime-base USF imaging.

Adopting QDs into the aforementioned USF CA protocols seemed impractical. Polymerization reaction or micelle preparation conditions might damage the passivation or surfactant layer of such nanocrystal, resulting in decreasing the long fluorescence lifetime. Alternatively, conjugating QDs to any carriers (polymer, nanocomposites, or micelle) would be more feasible, since water-soluble functionalized QDs are commercially available. The QDs-labeled conjugates were found actively fluorescent but lacking the long fluorescence lifetime of the QDs themselves. The conjugating reaction, such as NHS/EDC crosslinking, or/and purification (dialysis or centrifuge), might cause unexpected aggregation, resulting in the loss of long fluorescence lifetime.

Water-soluble QDs coated with streptavidin maintained a long fluorescence lifetime after binding with biotin-labeled DNA, and FRET was obviously observed (Saremi et al., 2014). This results encouraged us to adopt QDs into USF CA design for further lifetime-based imaging.

The development of USF CAs is still in the stage of verifying their functioning mechanism and communicating with the USF instrument, targeting CAs that were conjugated with biomolecules have not been reported yet.

### Mechanism of Ultrasound-Switchable Fluorescence Imaging

USF, also known as temperature-modulated fluorescence, uses ultrasound pulses to cause fluorophores in the ultrasound focus region to fluoresce, while fluorophores outside the focus are still in a state where no fluorescence is emitted. Based on the USF effect, the ultrasound focus is used to scan the biological tissue containing the USF chromophore labeled target and simultaneously detect the change of the fluorescence signal on the tissue surface. Through this procedure, eventually millimeter or even sub-millimeter resolution can be obtained of the fluorescence image at centimeter level depth (Cheng et al., 2014).

The process of ultrasonically switching or modulating the fluorophore to emit fluorescence is based on the temperature rise at the focal region of the ultrasonic wave, causing the microenvironment of the chromophore focal region to change (hydrophilic/hydrophobic environment, solvent polarity or viscous degree, etc.), and causes the chromophore to start emitting fluorescence. In practice, fluorophores with USF effects also emit weak fluorescence when there is no ultrasound. Using ultrasound, the quantum efficiency of fluorescence emission is enhanced, resulting in an increase in intensity or the lifetime of fluorescence emission (Cheng et al., 2014). Based on the changes in fluorescence characteristics caused by ultrasound, we can classify USF imaging modes into two categories: USF based on fluorescence intensity and USF based on fluorescence lifetime. The reported experimental results have shown that both imaging modalities have achieved deep tissue, high resolution imaging in biological tissue. We herein introduce relevant work from two imaging modes, fluorescence intensity-based USF and fluorescence lifetime-based USF.

### Fluorescent Intensity-Based USF Imaging Modality

**Figure 6:**
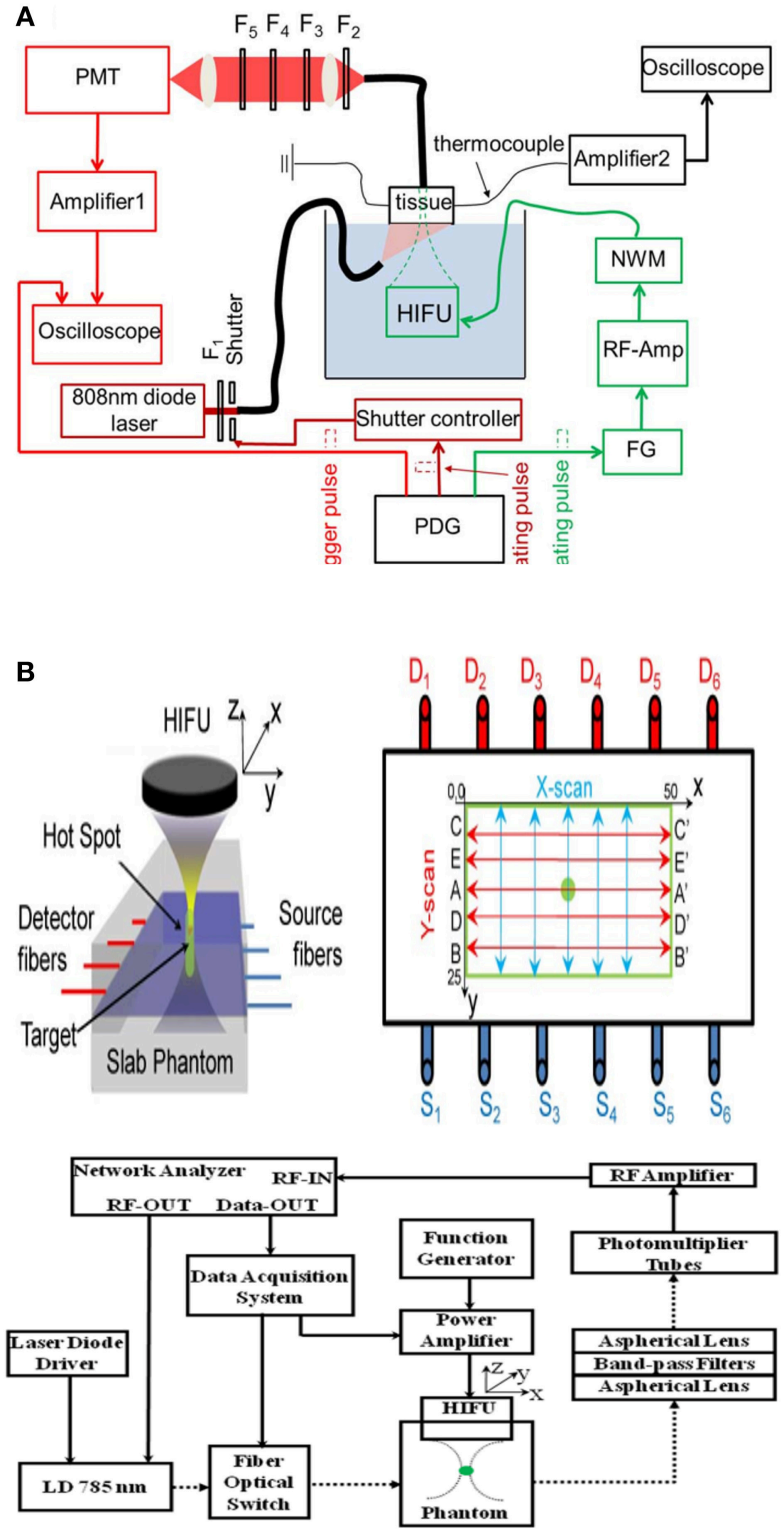
Diagrams of two fluorescence intensity-based USF imaging systems, reported by two different research groups: **(A)** Yuan et al., 2012; Pei et al., 2014; and **(B)** Lin et al., 2012a. Reproduced with permission. Copyrights 2012 API Publish and 2014^©^Springer Nature.

Figure 6A is a typical system diagram based on fluorescence intensity USF imaging (Pei et al., 2014). A continuous near-infrared laser beam emitted by a semiconductor laser at the wavelength of 808 nm is used as an excitation light to irradiate the surface of the biological tissue phantom. The silicone tubing in the biological tissue phantom is filled with USF CAs. The semiconductor laser continuously excites the tissue phantom while the system is operating. The chromophore emits fluorescence under the application of the excitation light, which propagates to the surface of the tissue phantom to be collected by the fiber bundle. The collected fluorescence passes through the filter and is converted into the electrical signal by the PMT and is displayed by the oscilloscope. The focus of HIFU scans the phantom point by point under the control of the translation stage. The fire and duration of ultrasound pulse, stepping of translation stage, and fluorescence signal acquisition are controlled by the pulse delay generator to obtain the USF signal. Finally, the simplest and most straightforward way to reconstruct a USF image is to use the USF signal for each pixel. According to the above principle, the key factor affecting the resolution of USF is the focal size of HIFU, and the factors affecting the imaging depth include the amplitude of fluorescence change caused by ultrasound and signal to noise ratio and sensitivity of the imaging system.

**Figure 7:**
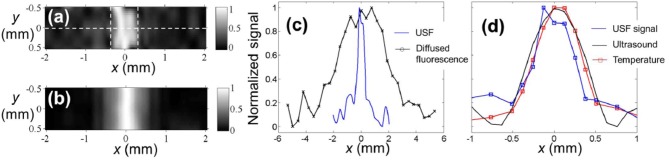
The USF image of the tube embedded into the porcine muscle tissue (Pei et al., 2014). ICG-encapsulated PNIPAM NPs were used as the contrast agents. The two dashed vertical lines represent the inner boundaries between the tube and USF contrast agents. **(b)** The diffraction-limited ultrasound image (C-mode) of the same tube in the tissue, obtained by raster scanning the same HIFU transducer on the x-y plane. **(c)** The profiles of the USF signal and the diffused fluorescence signal along the x axis at y = 0 (see the dashed horizontal line in **(a)**. **(d)** The profiles of the USF, ultrasound, and temperature signals along the x axis at y = 0. Both the USF and ultrasound image were normalized and interpolated based on a bicubic method. Reproduced with permission. Copyright 2014^©^Springer Nature.

Based on this system, the researchers imaged a silicone tube with an inner diameter of 0.69 mm in a 4 mm deep chicken tissue phantom, and they estimated that the imaging system had a point spread function of 0.29 mm (Figure 7). It should be noted that the lateral dimension of the focus of the HIFU used in this work is between 0.512 and 0.54 mm, so the above results actually exceed the acoustic diffraction limit. The mechanism for achieving the breakthrough of the acoustic diffraction limit is a non-linear acoustic effect, so that the size of the heating volume at the HIFU focus is smaller than the size of the sound pressure distribution (Yuan et al., 2013). Another possible reason of exceeding the sonic diffraction limit is the threshold effect of the USF chromophore. The ability to achieve resolution that exceeds the acoustic diffraction limit is one of the advantages of the USF imaging mechanism. The fluorescence chromophore used in this work has a maximum fluorescence variation of 9.1:1. Additionally, the technique of suppressing noise is not considered in the imaging system, therefore the imaging depth is only 4 mm.

**Figure 8:**
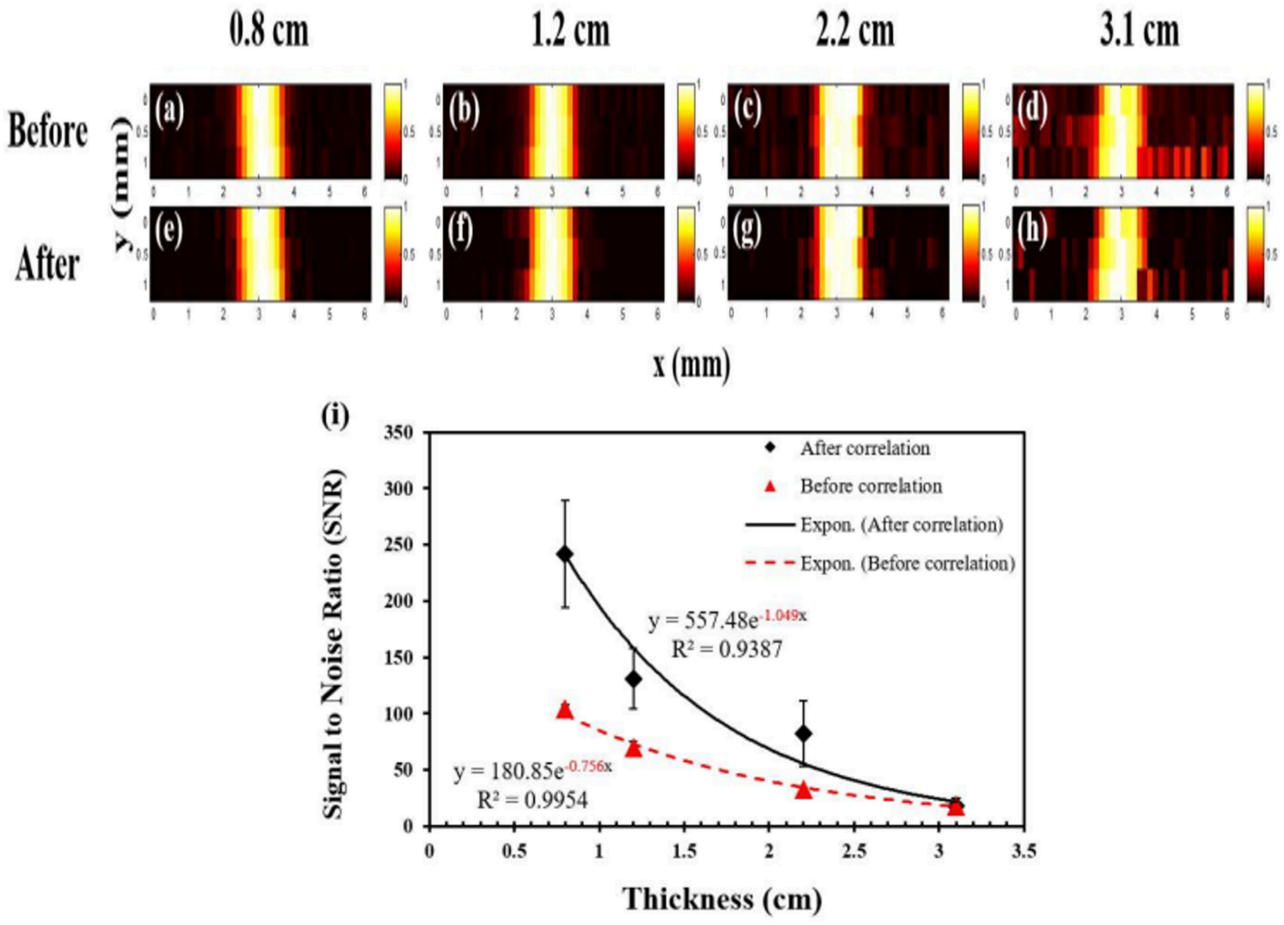
USF images of micro-tubes in porcine muscle tissue samples (Cheng et al., 2016). **(a–d)** The USF images of the micro-tubes (before correlation) that were embedded into pork muscle tissue samples with thicknesses of 0.8, 1.2, 2.2, and 3.1 cm, respectively. The I.D./O.D. of the tube is 0.31/0.64 mm; **(e–h)** The corresponding USF images processed by the correlation algorithm. **(a–h)** scales: X axis: 0-6 mm; Y axis: 0-1 mm. **(i)** The relationship between the SNR and the thickness of the sample before (triangles) and after (diamonds) the correlation processing (error bar: mean±standard deviation). Reproduced with permission. Copyright 2016^©^PLOS.

In 2016, the same group improved the USF imaging system using three aspects: (1) Loading the modulated signal on the excitation light and using the phase-locked amplification technique in fluorescence signal acquisition; (2) Correlating the signal with respect to the time domain characteristics of the USF signal identification; (3) Using better performing USF CAs, the fluorophore enhanced the fluorescence intensity by more than 200 times utilizing ultrasound (Cheng et al., 2016). Improved imaging system has a resolution of 900 μm in 3 cm deep biological tissue phantoms, see Figure 8.

**Figure 9:**
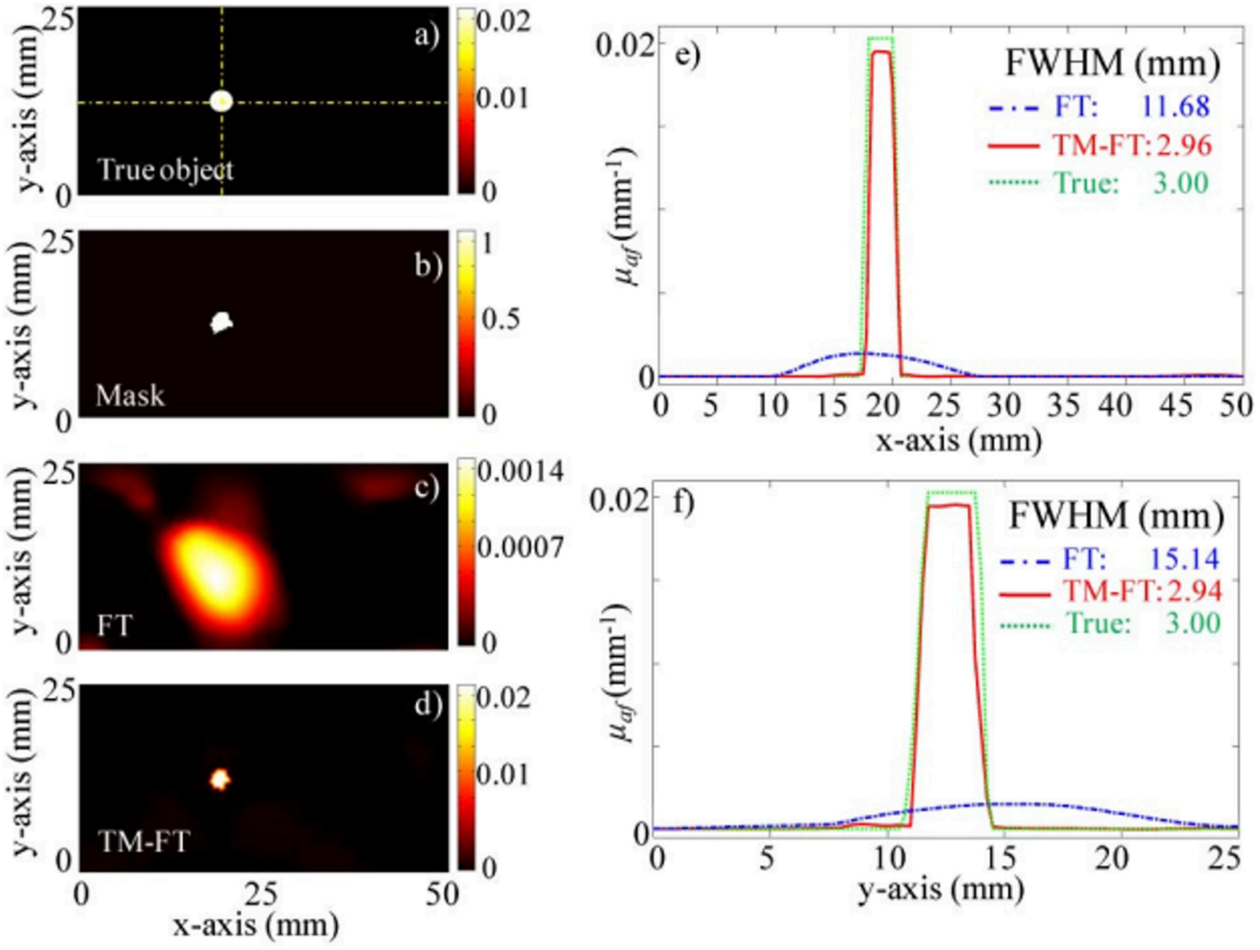
Experiment results for the 100 mm (*x*) × 40 mm (*y*) × 100 mm (z) agarose phantom with a single 3 mm diameter target (Nouizi et al., 2015). **(a)** True ThermoDot distribution in the optical fiber plane. **(b)** Binary map obtained using TM-FT. **(c)** Reconstructed fluorescence map using conventional FT with no a priori information. **(d)** Reconstructed fluorescence map using TM-FT. The profiles across the fluorescence target along **(e)** *x*- and **(f)** *y*-directions show that the size of the fluorescence target is accurately recovered. Copyright 2015^©^OSA Publishing.

In 2012, another research group also proposed the USF imaging mode (Lin et al., 2012a,b). In their first report on the USF, they achieved clear imaging of a biological tissue with a depth of 5 cm and a size of 3 mm. The resolution of the imaging system can reach 1.8 mm. The imaging system used by the Lin team is shown in Figure 6B. In this system, the HIFU has a focus size of 1.33 mm. Since the HIFU focus is required to scan the tissue phantom point by point, it takes more than 1 hour for the imaging system to acquire an image of 8 × 8 mm^2^. In 2015, they used the HIFU continuous scanning method to increase the system imaging speed by 40 times without reducing the imaging resolution, see Figure 9 (Nouizi et al., 2015). In the numerical simulation in 2015 and the experimental work in 2017, the same group reconstructed the image of the fluorophore concentration distribution using the fluorescence tomographic image reconstruction algorithm and introduced the USF signal as the constraint condition equation (Lin et al., 2015; Kwong et al., 2017a,b). The reconstructed USF image has a deviation of <12% in chromophore absorption, while the chromophore absorption deviation of the fluorescence tomographic image is larger than 90%.

In a more recent work published in 2017 (Kandukuri et al., 2017), a dual-modality imaging system includes USF and B-mode ultrasound were built, by which the USF multi-color image and structure image were acquired. With two 90°-crossed HIFU transducers, the same resolution for axial and lateral direction were achieved. In the latest work (Yao et al., 2019), the optical detector of USF system was changed from PMT to CCD and the scanning was along z-shape path. The imaging time was improved by 4 times over the raster scan, and the USF imaging was validated by a micro-CT system.

### Fluorescent Lifetime-Based USF Imaging Modality

In fact, the first article about USF imaging published in 2012 was based on the increase of the fluorescence lifetime of the USF CAs under HIFU (Yuan et al., 2012). The imaging system uses a picosecond pulsed laser as the excitation light. Under ultrasound, the temperature at the focus of the sound field rises from room temperature by 8° to 32°C, and the fluorescence lifetime of the USF CAs changes from about 4 nanoseconds to about 14 nanoseconds. Therefore, by detecting whether there is a fluorescent photon after a specific delay time with respect to the excitation light pulse, it is possible to determine whether or not the USF signal is present. Based on this principle, the authors scanned a silicone tube with a size of about 180 μm with a depth of about 20 mm in a biological tissue. The resulting image had a FWHM of about 260 μm. The HIFU used in this system has a focal size of 420 microns. This work initially demonstrated the ability to perform sub-millimeter-resolution deep tissue imaging based on the USF mechanism. In fact, fluorescence lifetime-based USF imaging should have a higher signal-to-noise ratio than fluorescence intensity-based USF imaging, making this imaging mode is more attractive. However, an important issue that needs to be addressed in this work is that the USF chromophore used has an excitation wavelength of about 475 nm. For the study of biological tissues, especially living tissues, red or near-infrared-sensitive USF fluorophores is a more suitable wavelength for the USF imaging.

Unlike the above-mentioned time-domain USF system, some researchers built the USF system in the frequency domain in 2012 to simultaneously image fluorescence intensity and lifetime for targets with USF CAs (Lin et al., 2012a). This work imaged a target with a depth of 2 cm and a size of 3 mm in the biological tissue, and the FWHM of the target on the image was 3.2 mm.

## Comparison With PAI

In PAI, biological tissue is excited by laser or radio frequency electric-magnetic pulses, and some molecules in the tissue transition into the excited state. Subsequently, the absorbed photon energy is released partially or completely through non-radiative transitioning in the form of heat in picoseconds to nanoseconds, resulting in an increase in local tissue temperature. The increase of local temperature induces the pressure to generate the photoacoustic waves propagating out of the tissue. The photoacoustic wave is detected by an ultrasonic transducer and collected by a computer. Finally, the signal is processed to obtain a photoacoustic image. Based on the principles above, it can be concluded that the photoacoustic image gives the optical absorption distribution of a particular chromophore in the tissue. Because certain molecules in a living organism, or CA distributed in a biological tissue, can reflect its molecular information at the specific wavelength or bandwidth, the photoacoustic modality can produce images of the structural, functional, physiological, and pathological processes of an organism.

Compared with UMF and USF, the development of PAI is more sophisticated. There have been a large number of review papers presenting the current state of PAI research. The main technical features and development of PAI are briefly described here as a reference for reviewing UMF and USF. The photoacoustic effect was first discovered by Alexander G. Bell in 1880. In 1994, Kruger et al. began imaging the interior of biological tissue phantoms based on photoacoustic effects (Kruger, 1994; Kruger and Liu, 1994). For the first 10 years since its inception, researchers typically used a single ultrasound probe to receive photoacoustic signals, where B-mode imaging was obtained and 3D imagings were performed using point-by-point scanning (Hoelen et al., 1998; Geng and Wang, 2000; Wang et al., 2003). Ultrasound probes typically operate at frequencies of a few megahertz. Therefore, at this stage, the resolution of photoacoustic imaging is usually above 100 microns. Even though the resolution is not very high, the imaging depth is on the centimeter scale, which has shown great advantages in deep tissue imaging. In the past 10 years, with the development of ultrasonic detection devices and excitation light sources, photoacoustic imaging has made rapid progress (Wang and Yao, 2016). The frequency and bandwidth of the ultrasonic detector increased from megahertz to hundreds of megahertz, and the resolution of the corresponding photoacoustic imaging reaches the order of tens of microns (based on optical means, the resolution can be increased below the optical wavelength). Ultrasound probes from a single ultrasound transducer to an ultrasound transducer array avoid mechanical scanning and increase imaging speed to meet video needs. Excitation sources, evolving from initial pulsed xenon lamps and YAG lasers to continuous tunable parametric oscillators, laser diodes, and light-emitting diodes, enable miniaturization and portability of multi-wavelength imaging systems. Therefore, photoacoustic imaging has realized two basic working modes of photoacoustic computed tomography and photoacoustic microscopy, enabling photoacoustic imaging with resolutions of several hundred micrometers, tens of micrometers, micrometers and submicron scales, and even exceeding the optical diffraction limit. Of course, the increase in resolution is at the expense of imaging depth. The imaging depth of photoacoustic computed tomography can reach about 5 cm, while the imaging depth of an acoustic resolution photoacoustic microscope has been reduced to about 1 cm or less.

The rapid development of photoacoustic technology has driven preclinical research and clinical application, mainly focusing on breast cancer imaging and blood oxygen concentration imaging (Upputuri and Pramanik, 2016). Moreover, companies such as FUJIFILM VisualSonic and iThera Medical have successively launched commercial PAT systems.

As shown in Table 3, five parameters, working mode, resolution, penetration depth, depth-to-resolution ratio, and frame rate, were selected to make a straightforward comparison among PAI, UMF, and USF methods. The performance of USF had impressive results, with a resolution of ~100 μm and penetration depth of <50 mm, which is comparable with conventional PAT. Such promising results make researchers believe that USF will surpass PAT in the future, although frame rates and depth-to-resolution ratio of USF are still under optimization.

**Table 3:** Comparison of selected specifications of PAI, UMF, and USF imaging systems.

**Imaging modality**	**Working mode**	**Resolution**	**Penetration depth**	**Depth-to-resolution ratio**	**Frame rate**
PAI	OR-PAM	lateral 0.22μm axial 30μm	~1 mm	~200	Visual rate could be satisfied
	AR-PAM	≥7 μm	~10 mm		
	PAT	~100 μm	<70 mm		
UMF	Intensity or lifetime	~1 mm	~1 mm	~1	NR
USF	Intensity mode	~100 μm	<50 mm	~10	~10 mins
	Lifetime mode				

Although the imaging indicators of UMF and USF are different from PAI, their key imaging capabilities are not comparable to PAI. For example, targeted imaging for life function is based on targetable CAs. This is an advantage inherited from fluorescence imaging. Therefore, there is a need to advance the mechanisms of these two imaging modalities, systems, animal experiments, and CAs. Extrinsic CAs with UMF or USF signals are key to the study of these two imaging modalities. Most PAI imaging studies do not rely on extrinsic CAs, which use only intrinsic absorption imaging of biological tissue. External CAs are an important option only when PAI needs to further increase imaging depth, improve other performance or enable specific imaging function. However, the work of the UMF or USF imaging mode always requires a special function of the external CA. CAs are one of the biggest challenging factors in the development of these two imaging modalities. Because the study of CAs applied to these two imaging modalities is still ongoing, no commercial products are available. This situation limits the amount of collaboration other researchers can have in joining the development of these two imaging modalities. Additionally, CAs that work in one environment may no longer work in another environment, the mechanisms and characteristics of the work may change, or overall performance may degrade. This can affect the advancement of imaging research from tissue phantoms to animal experiments and even clinical studies. The working mechanism and performance studies of the imaging devices and imaging systems required for imaging are another key point in UMF or USF imaging model research. The imaging system's operating mode (currently primarily transmissive mode) needs to be extended, and key imaging metrics such as signal-to-noise ratio, resolution, imaging depth, and speed need to be improved from a system perspective. Developments in imaging devices such as ultrasound transducers would also very beneficial; for example, if an array of ultrasound transducers were available, the imaging speed would be greatly improved.

## Summary and Outlook

The idea to create a new combination with known materials to fulfill the requirements of UMF or USF CAs is intriguing and encouraging. Two remarkable steps have been made: (1) the conception of designing unique CAs and of their subsequent synthesis and characterization, and (2) the accomplishment of producing promising results of using CAs in bio-imaging instruments, reaching a proof-of-concept landmark. As for UMF CAs, microbubbles have become a perfect carrier and mediator for loading UMF CAs. Using microbubble-based CAs, researchers are closing in on unfolding the myth of UMF bioimaging mechanism. Numerous designs and testing had been documented for the development of USF CAs. NIR dye-encapsulated PINIPAM nanocomposites were considered as the first class of USF CAs (USF CAs No.1), as it demonstrated better performance properties over polymer-based CAs. Great improvement in shelf-life (>6 months) was obtained by switching the surfactant from SDS to Pluronic polymer. An ADP-CA dye that is extremely sensitive to micro-environment gave the researchers an opportunity to develop a USF CAs No.2. However, the dye is incompatible in the previous synthesis protocol due to its poor water solubility. Therefore, the ADP-CA-encapsulated micelle was developed and demonstrated significant advantage in fluorescence intensity ratio in comparison to that of ICG-based USF CAs No.1, but the ADP-CA's emission wavelength is shorter than ICG. Two classes of USF CAs were successfully integrated into the newly-built USF bio-imaging system, providing convincing intensity-based USF imaging results in tissue.

With excitement in such remarkable progress in UMF and USF bioimaging, we need to keep in mind there is still a long way to go to make them to be comparable to PAI. Fluorescence lifetime-based USF may be one of the solutions for USF bioimaging to stride forward. The key is the development of life-time USF CAs, which is still challenging, because of the limited availability of long lifetime NIR fluorophore and the complications in adopting any of them into the two established protocols. Water-soluble QDs might be a promising candidate, along with sophisticated bio-affinity conjugation.

## Author Contributions

YP and M-YW contributed to this work equally. Specifically YP wrote the section of bio-imaging instrument and M-YW wrote the section of contrast agents. YP and M-YW reviewed the article.

### Conflict of Interest Statement

The authors declare that the research was conducted in the absence of any commercial or financial relationships that could be construed as a potential conflict of interest.

## Footnotes

^1^https://www.accessdata.fda.gov/scripts/cder/drugsatfda/index.cfm
